# Expression of indoleamine 2,3-dioxygenase in nasopharyngeal carcinoma impairs the cytolytic function of peripheral blood lymphocytes

**DOI:** 10.1186/1471-2407-9-416

**Published:** 2009-11-30

**Authors:** Peng Liu, Bai-Lu Xie, Shao-Hui Cai, Yun-Wen He, Ge Zhang, Yan-Mei Yi, Jun Du

**Affiliations:** 1 Department of Microbial and Biochemical Pharmacy, School of Pharmaceutical Sciences, Sun Yat-sen University, No 132 Waihuandong Road, University Town, Guangzhou 510006, China; 2 College of Pharmacy, Jinan University, Guangzhou 510632, China

## Abstract

**Background:**

Tumor-specific cytotoxic T cells and infiltrating lymphocytes are frequently found in tumor tissues in patients with nasopharyngeal carcinoma (NPC). Most patients with NPC, however, especially those with advanced stages, have a poor clinical prognosis despite conventional immunotherapy. The aim of this work was to examine the effect of indoleamine 2,3-dioxygenase (IDO), an immunosuppressive enzyme, on the lymphocyte function in NPC.

**Methods:**

The NPC cell line CNE2 was treated by interferon-γ (IFNγ) and the levels of IDO expression was analyzed by Western blotting and reverse phase high-performance liquid chromatography (HPLC). Lymphocytes from health human exposed to the milieu created by IDO-positive CNE2 cells and the lymphocyte cytotoxicity to target tumor cells was analyzed by standard lactate dehydrogenase (LDH) release assay. Additionally, expression of IDO was determined by Immunohistochemical assay in the tumor tissues form clinically evaluated NPC.

**Results:**

IDO expression was acutely induced in the NPC cell line CNE2 by low dose interferon-γ (IFNγ) or by co-incubation with activated lymphocytes. Exposure to the milieu created by IDO-positive CNE2 cells did not promote lymphocyte death, but lymphocyte cytotoxicity against target tumor cells was impaired. The suppression of lymphocyte cytotoxic function was fully restored when the conditioned medium was replaced by fresh medium for 24 h. In additionally, the IDO-positive cells were found scattered in the tumor tissues from patients with NPC.

**Conclusion:**

Altogether, these findings suggest that IDO-mediated immunosuppression may be involved in the tumor immune evasion, and that blocking IDO activity in tumor cells may help to re-establish an effective anti-tumor T cell response in NPC.

## Background

Nasopharyngeal carcinoma (NPC) is an Epstein-Barr virus (EBV)-associated malignancy with high prevalence in Southern China and Southeast Asia [1]. Guangdong province, also called Canton, has the highest prevalence, earning NPC the name of 'Canton tumor'. Due to the non-specific nature of the nasal and aural symptoms and the difficulty of making a clinical examination of the nasopharynx, most patients with the disease are diagnosed only when the tumor has reached an advanced stage (stages III and IV) [2]. Radiotherapy is the main treatment for this disease, but patients with intermediate and advanced stages who only receive radiotherapy have a 5-10-year survival rate of only 40%. Hence, novel approaches to the treatment of NPC are needed to improve the prognosis of patients with NPC.

Immunotherapeutic strategies aimed at boosting anti-tumor immunity are promising candidates for the treatment of NPC. A few studies have focused on reversing the impaired immune response to NPC tumors [3]. Determination of the mechanisms behind the dysfunction of cytotoxic T lymphocytes in patients with NPC would undoubtedly be of help in the development of optimal immunotherapeutic strategies for NPC. It has been reported that cytokine expression in tumor infiltrating lymphocytes (TILs) in NPC patients is comparable to that in healthy controls. Interferon-γ (IFNγ) is one of the prominent cytokines associated with immune activation and immunosuppression [4].

IFNγ, also called type II interferon or immune interferon, is mainly produced by activated T cells and NK cells, and acts as an important mediator of the immune system, involving activities such as immuno-modulation, lymphocyte recruitment and activation, anti-pathogen and anti-tumor activity [5]. Although IFNγ was first used to treat patients with NPC in 1987 [6], there was no further report on IFNγ therapy for NPC since 1993 due to some cases were shown to be unresponsive. In most cases of NPC, the dense infiltration of lymphocytes is observed in the tumor site, and EBV-associated viral antigens in tumor cells are presented for lymphocyte recognition, nevertheless IFNγ fails to exert its intended anti-viral and anti-tumor effects in the patients with NPC [7,8].

IFNγ has the specific ability to induce indoleamine 2,3-dioxygenase (IDO) expression in various kinds of tumors [9]. IDO is responsible for initiating the first, rate-limiting step in tryptophan metabolism in the kynurenine (Kyn) pathway [10]. Growing evidence suggests that IDO-mediated tryptophan metabolism in antigen presenting cells and tumor cells represent a vital mechanism for potential T cell suppression during tumor growth. Localized tryptophan deficiency and the accumulation of toxic metabolites in tumor-draining lymph nodes and the tumor microenvironment could contribute to the growth arrest, inactivation, and even death of T cells [11-13]. IDO has been investigated in cervical, colorectal, hepatocellular, ovarian carcinoma, endometrial cancer and thyroid cancer [14-18], but to our knowledge, no detailed studies have investigated the expression of IDO in NPC.

We therefore aimed to examine the roles of IFNγ and IDO in NPC, in order to throw new light on the mechanism by which immune evasion affects therapeutic treatment of NPC.

## Methods

### Cell culture

The human nasopharyngeal carcinoma cell line CNE2 was established at Hunan Medical College, China [19,20]. CNE2 cells used in this study were maintained in our laboratory in RPMI 1640 medium (Gibco-BRL, MD) supplemented with 10% fetal calf serum (FCS, PAA Laboratories, MA) and antibiotics (100 U/ml penicillin, 100 μg/ml streptomycin). When the monolayer of CNE2 cells reached 60% confluency, IFNγ (China National Biotec Group, Shanghai, China) was added to the medium at 0-500 U/ml, for the indicated time.

Human peripheral blood was donated by healthy volunteers, following appropriate informed consent. Peripheral blood mononuclear cells (PBMCs) were isolated by density gradient centrifugation through Ficoll-Hypaque (Tian Jin Hao Yang Biological Manufacture Co., TianJin, China) according to standard procedures. Monocytes and phagocytes were removed by adherence to plastic by culturing PBMCs in RPMI 1640 containing 50 U/ml exogenous interleukin-2 (IL-2, Kexing Biotech Co., Shenzhen, China) for 4-5 h. The non-adherent peripheral blood lymphocyte (PBL) fraction was harvested and cultured at a density of 2 × 10^6 ^cells/ml in 24-well plates with complete RPMI 1640 medium (containing 10% FCS, 100 U/ml penicillin and 100 μg/ml streptomycin). IL-2 100 U/ml was added to the medium every other day, for an incubation period of at least 4 days.

### Conditioned medium treatment

CNE2 cells were cultured in 6-well plates (3 × 10^5 ^cells/well) in the absence or presence of 50 U/ml IFNγ for 12 h, and the medium was then replaced by fresh medium, with or without 100 μM 1-methyl-D/L-tryptophan (1 MT, Sigma-Aldrich, MO). Twenty-four hours after medium replacement, culture medium was harvested as conditioned medium (CM) from CNE2 cells (CNE2-CM), and used for the incubation of PBLs.

To prepare the CM from activated PBLs for treating CNE2 cells, IL-2-treated PBLs were suspended in complete RPMI 1640 medium or CNE2-CM, and all media were supplemented with 100 U/ml IL-2. After culture for 24 h, PBLs were harvested for cytotoxicity assays and the supernatant was collected and used for incubating CNE2 cells. CNE2 cells were first cultured in complete RPMI 1640 medium for 18 h, and the medium was then replaced by the above PBL CM and the CNE2 cells were incubated for 24 h.

### Immunohistochemical assay

Formalin-fixed and paraffin-embedded NPC samples (n = 9) were selected from archival material from the Department of Pathology in Sun Yat-sen University of Medical Sciences. The sections were deparaffinized in xylene and rehydrated through graded alcohol washes to distilled water. Endogenous peroxidase was blocked with 0.3% H_2_O_2 _for 30 min and non-specific staining was prevented by incubation in commercial goat serum (Wuhan Boster Biological Technology Ltd, Wuhan, China). Sections were stained using the streptavidin-peroxidase (SP) staining method by incubation with rabbit anti-human IDO polyclonal antibody (1:500, generated in our laboratory [21]) at room temperature for 1 h. After washing in phosphate buffered saline (PBS) containing 0.05% Tween-20 (PBS-T), the sections were incubated with secondary antibodies from the EnVision™ Detection Kit (Gene Tech, Shanghai, China). Slides were stained in the absence of primary antibodies to evaluate non-specific secondary antibody reactions and were counterstained with hematoxylin.

In order to detect the expression of IDO in CNE2 cells they were immunohistochemically stained using the same SP staining method as for the clinical specimens.

### Western blot analysis

The treated CNE2 cells were lysed in cell lysis buffer (50 mmol/l Tris-Cl, pH = 8.0; 150 mmol/l NaCl; 0.02% NaN_3_; 0.1% sodium dodecyl sulfate (SDS); 1% NP-40; 0.5% sodium deoxycholate; 1 mol/l EDTA). The lysates were cleared by centrifugation and denatured by boiling in Laemmli buffer. Equal amounts of protein (20 μg/well) were separated by 10% SDS-polyacrylamide gel electrophoresis and transferred to polyvinylidene difluoride membranes (Bio-Rad, CA). After blocking in Tris-buffered saline containing 0.1% Tween-20 and 5% nonfat milk, the membrane was incubated with the rabbit anti-human IDO polyclonal antibody, followed by incubation of horseradish peroxidase-conjugated anti-rabbit IgG (Wuhan Boster Biological Technology Ltd). Protein was visualized by enhanced chemiluminescence (ECL, Pierce, IL), For the loading control, β-actin (1:10,000, Beijing Biosynthesis Biotechnology Co., Beijing, China) was probed with the primary antibodies after the membrane was stripped of the IDO antibodies using stripping buffer.

### Measurement of IDO activity

The culture medium for IDO activity analysis was mixed with 1/4 volume of 30% trichloroacetic acid and then centrifuged at maximum speed for 20 min at 4°C to precipitate the protein. The concentration of Kyn in the culture medium was measured by reverse phase high-performance liquid chromatography (HPLC), using a Waters Alliance HPLC system (Waters, MA). Kyn was detected by the UV detector at 340 nm and the results were processed in Breeze version 3.30 SPA. IDO activity was determined by the specific Kyn production after subtracting the non-specific Kyn concentration in the RPMI 1640 medium with 10% FCS.

### Flow cytometric analysis

PBLs untreated and pre-treated with IL-2 were plated into 24-well plates in complete RPMI 1640 medium, or CNE2-CM without 1 MT (described in 2.2). After 48 h incubation, cells were harvested, washed with PBS, and resuspended in annexin V-binding buffer. Fluorescein isothiocyanate-conjugated annexin V (Trevigen, MD) was added to a final concentration of 100 ng/ml and cells were incubated in the dark for 15 min at room temperature. Propidium iodide (PI, Trevigen) was added to each sample so that the final concentration of PI was 50 mg/ml before flow cytometric analysis.

### Cytotoxicity assay

The cytotoxic activity of the PBLs was determined by standard 4 h lactate dehydrogenase (LDH) release assay using CytoTox 96^® ^Non-Radioactive Cytotoxicity Assay (Promega, WI) in U-bottomed 96-well microplates. To obtain the target cells, CNE2 cells were seeded at a density of 5 × 10^3 ^cells/well in U-bottomed 96-well microplates, cultured for 18 h, and the medium was then replaced by 50 μl fresh phenol red-free RPMI 1640 medium (Gibco-BRL) containing 5% FCS. To prepare the pre-treated effector cells, IL-2-stimulated PBLs were incubated for 24 h in CNE2-CM and then collected and resuspended in phenol red-free RPMI 1640 containing 5% FCS. Fifty microliters of the suspensions were added to each well at various indicated effector:target (E:T) ratios. After 4 h incubation, the plates were centrifuged and 50 μl of the supernatant was transferred to new 96-well flat-bottomed plates, following the manufacturer's instructions. The release of LDH into the supernatant was quantified by recording the absorbance at 490 nm. The percentage of cytotoxicity was calculated as follows:

## Results

### The expression of IDO in the NPC cell line CNE2 is highly sensitive to IFNγ stimulation

**Figure 1:**
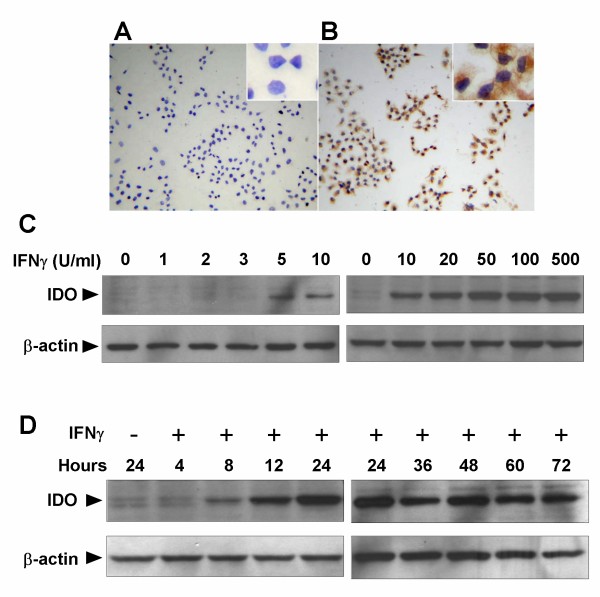
**Effect of IFNγ on indoleamine 2,3-dioxygenase (IDO) expression in the nasopharyngeal carcinoma cell line, CNE2**. **(A) and (B) IFNγ-induced IDO protein expression in CNE2 cells assessed by immunohistochemistry**. CNE2 cells were treated with (A) or without (B) 50 U/ml IFNγ for 24 h, and stained with anti-IDO antibody. The images were captured at ×100 magnification, and ×400 magnification for inserts. (C) and (D) IFNγ induced IDO protein expression in CNE2 cells in a dose- and time-dependent manner. CNE2 cells were cultured in the presence of indicated concentrations of IFNγ for 24 h (C), CNE2 cells were cultured with 50 U/ml IFNγ for the indicated incubation time (D). Cells were harvested and IDO expression was analyzed by western blotting using anti-IDO antibodies. Data are representative of three individual experiments.

We investigated the effect of IFNγ, a well-known potent IDO inducer, on the IDO expression in the NPC cell line CNE2. As shown in Fig. 1A and 1B, immunohistochemical assay identified IDO expression in the cytoplasm of CNE2 cells treated with IFNγ, whereas no IDO expression was found in untreated CNE2 cells. We performed western blot analysis to investigate the effects of varying concentrations and incubation periods of IFNγ on the expression of IDO in CNE2 cells. As shown in Fig. 1C, treatment with low dose IFNγ (5 U/ml) induced IDO expression, which was further increased in an IFNγ concentration-dependent manner. When CNE2 cells were treated with 50 U/ml IFNγ, expression of IDO was observed within 8 h, peaked at 24 h, and then remained unchanged for at least 72 h (Fig. 1D). These results suggest that IDO expression in NPC cells was an inducible event that was highly sensitive to IFNγ stimulation

### Enzymatic activity of IDO is up-regulated by IFNγ stimulation in the NPC cell line CNE2

**Figure 2:**
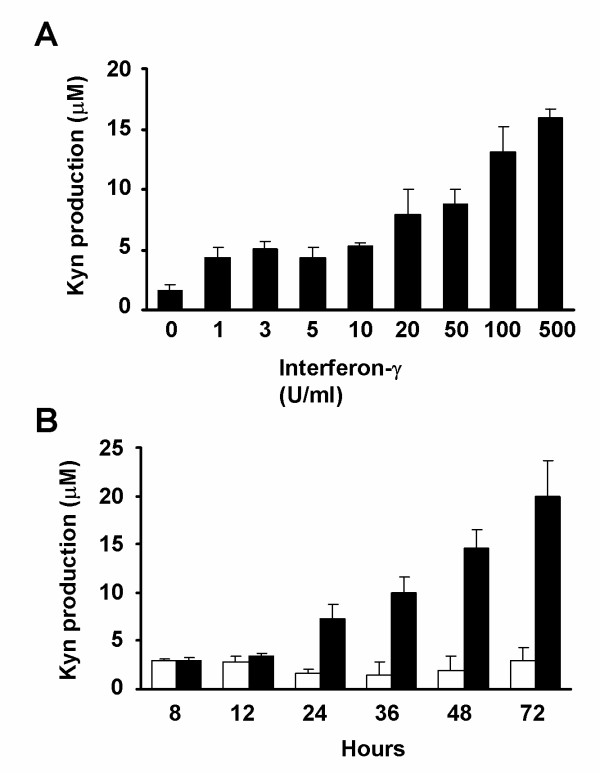
**IFNγ induced indoleamine 2,3-dioxygenase activity in CNE2 cells in a dose- and time-dependent manner**. (A) After CNE2 cells were cultured in the presence of different concentrations of IFNγ for 24 h, the supernatants of CNE2 cells were analyzed for Kyn production using high performance liquid chromatography (HPLC). (B) CNE2 cells were cultured with 50 U/ml IFNγ for different incubation times and the supernatants were analyzed for Kyn production by HPLC. □: untreated groups; ■: treated groups. Each value is the mean ± SD of three determinations.

Some recent studies have demonstrated a discrepancy between IDO expression and its enzymatic activity [22,23], and we therefore investigated the enzymatic activity of IDO in NPC cells. Kyn production was measured as an indicator of IDO activity [24] in CNE2 cells at varying concentrations and incubation periods of IFNγ, using reverse phase-HPLC. CNE2 cells were first treated with different concentrations of IFNγ for 24 h. Kyn production was undetectable in the culture medium of untreated cells, but was significantly increased by IFNγ treatment in a dose-dependent manner (Fig. 2A). CNE2 cells were then treated with 50 U/ml of IFNγ for 0-72 h and Kyn production was increased in a time-dependent manner, after treatment for 24 h (Fig. 2B), whereas basal levels were maintained in the untreated cells. The HPLC results indicated that the enzymatic activity of IDO in CNE2 cells treated with IFNγ was enhanced in a dose- and time-dependent manner.

### Activated PBLs induce expression of IDO in the NPC cell lineCNE2

**Figure 3:**
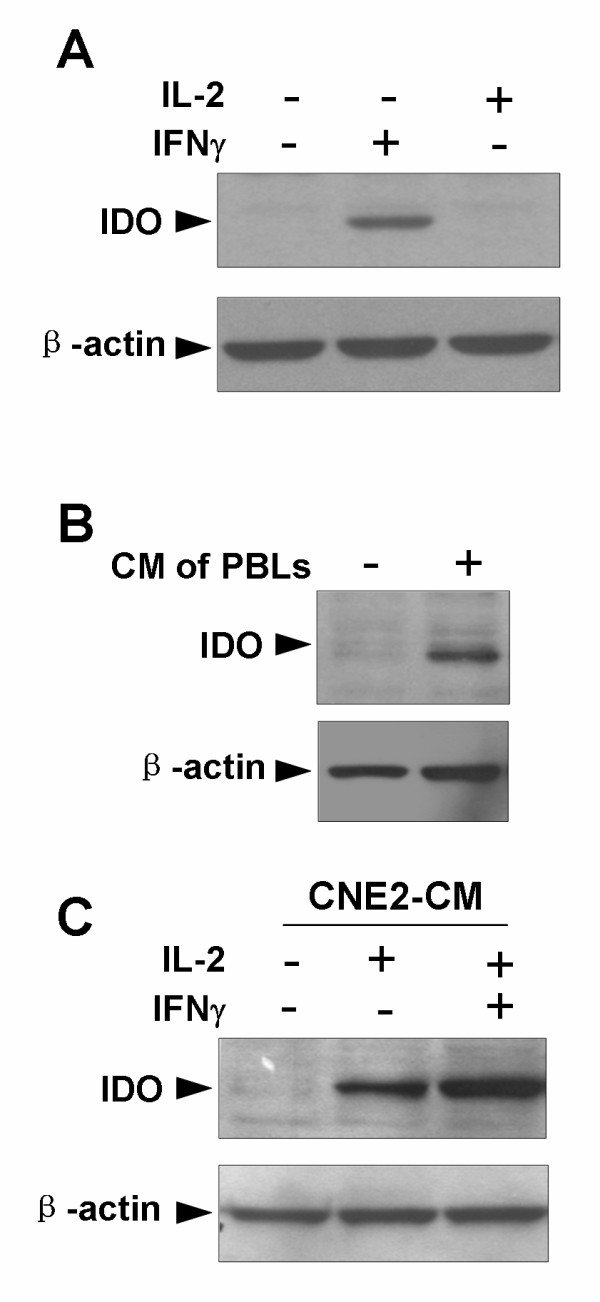
**Western blotting analysis to detect indoleamine 2,3-dioxygenase (IDO) expression in the nasopharyngeal carcinoma cell line CNE2 induced by activated peripheral blood lymphocytes (PBLs)**. (A) CNE2 cells were treated with or without 100 U/ml IL-2 or 50 U/ml IFNγ for 24 h. (B) PBLs were stimulated with 100 U/ml IL-2 for 96 h, washed to remove early generated cytokines and cultured in fresh complete RPMI 1640 medium supplemented with 100 U/ml IL-2 for 24 h. The PBL supernatant was then used as the CM to incubate CNE2 cells for 24 h. (C) PBLs were stimulated with 100 U/ml IL-2 for 96 h and washed to remove early generated cytokines. After further incubation with IL-2-supplemented CNE2-CM from CNE2 cells treated or untreated with IFNγ for 24 h, the PBL supernatants were collected and used to incubate CNE2 cells for 24 h. Expression of IDO in CNE2 cells was detected by western blotting analysis with anti-IDO antibodies. The data are representative of three individual experiments. CM, conditioned medium; CNE2-CM, conditioned medium derived from CNE2 cells.

Since TILs at tumor sites in NPC patients appear to be in an activated state [25] and may be involved in inducing IDO expression, we mimicked the interplay between activated lymphocytes and tumor cells by culturing CNE2 cells in CM from IL-2 stimulated PBLs, and analyzed the amount of IDO expression by western blot analysis. In order to establish if IL-2 was able to directly induce the expression of IDO, CNE2 cells were incubated in complete culture medium supplemented with 100 U/ml IL-2 for 24 h. Western blot analysis demonstrated that IL-2 in the culture medium failed to induce IDO expression in CNE2 cells (Fig. 3A). To investigate the effect of activated lymphocytes on IDO expression, CM from PBLs stimulated by IL-2 was harvested and used to incubate CNE2 cells. IDO expression was observed in CNE2 cells after incubation with CM from PBLs for 24 h (Fig. 3B).

IDO expression in NPC cells would alter the microenvironment by depletion of tryptophan and production of Kyn, so potentially affecting the ability of PBLs to induce IDO expression. To explore this possibility, PBLs were stimulated with 100 U/ml IL-2 for 96 h and washed to remove early generated cytokines. After further incubation with IL-2-supplemented CNE2-CM from CNE2 cells treated or untreated with IFNγ for 24 h, the PBL supernatants were collected and used to incubate CNE2 cells for 24 h. Western blot analysis showed that, as with CM prepared by incubating PBLs with CNE2-CM derived from untreated CNE2 cells, CM from PBLs which had been cultured in CNE2-CM derived from IFNγ-treated CNE2 cells was also able to induce IDO expression (Fig. 3C).

### Exposure to the microenvironment created by IDO-positive CNE2 cells does not severely reduce PBL survival

**Figure 4:**
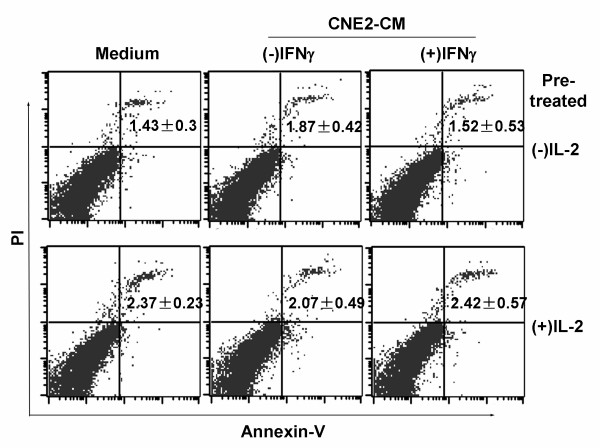
**Exposure to the conditioned medium derived from indoleamine 2,3-dioxygenase (IDO)-positive CNE2 cells did not induce PBL death**. PBLs were pre-treated with or without IL-2 for 96 h and then cultured in CNE2-CM derived from IFNγ-treated or untreated CNE2 cells for a further 48 h. Cell death was detected by flow cytometric analysis, using annexin V and propidium iodide as indicators. CNE2-CM, conditioned medium derived from CNE2 cells.

Several reports have suggested that T cells are highly susceptible to cell death in an IDO-expressing microenvironment [26,27], and we therefore determined if exposure to CM from IFNγ-treated CNE2 cells reduced PBL survival. PBLs were pre-treated with or without IL-2 for 96 h and then cultured in CNE2-CM derived from IFNγ-treated or untreated CNE2 cells for 48 h. Cell death was detected by flow cytometric analysis, using annexin V and PI as indicators. As shown in Fig. 4, in the IL-2-untreated groups, PBLs unexposed to CNE2-CM, which served as the control, showed a basal rate of cell death of 1.4 ± 0.3%, and that rate was unaffected by exposure to CNE2-CM derived from IFNγ-treated or untreated CNE2 cells. A similar result was observed in the IL-2-treated groups, where the death rate of PBLs exposed to CNE2-CM derived from IFNγ-treated or untreated CNE2 was the same as that of the control.

### Exposure to conditioned medium derived from IDO-positive CNE2 cells impairs cytolytic activity of PBLs

**Figure 5:**
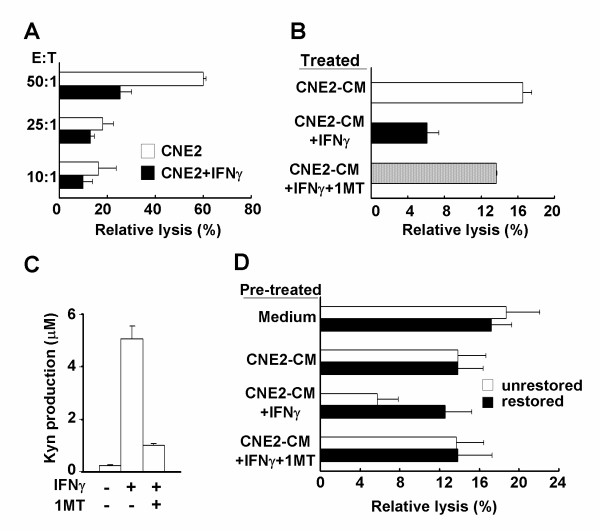
**Cytolytic activity of peripheral blood lymphocytes (PBLs) was impaired by incubation with CNE2-CM from indoleamine 2,3-dioxygenase-positive CNE2 cells**. (A) Activated PBLs were cultured with CNE2-CM from CNE2 cells treated with or without 50 U/ml IFNγ for 24 h and cytolytic activity against CNE2 cells was evaluated using a standard lactate dehydrogenase (LDH) release assay, as described in Materials and Methods. The effector:target (E/T) ratios are indicated. (B) Activated PBLs were cultured with CNE2-CM from CNE2 cells treated with or without 50 U/ml IFNγ and/or 100 μM 1 MT for 24 h, and cytolytic activity was evaluated using the LDH release assay. The E/T ratio was 10:1. (C) CNE2 cells were treated with or without 50 U/ml IFNγ and/or 100 μM 1 MT for 24 h. The CNE2 cell supernatants were analyzed for kynurenine production by high performance liquid chromatography, as described in Materials and Methods. (D) Activated PBLs were incubated with CNE2-CM from IFNγ-treated or untreated CNE2 cells containing 100 U/ml IL-2 for 96 h, and restored groups were transferred to IL-2-supplemented fresh medium for 24 h, the cytolytic activity of each group was analyzed by LDH release assay. The E/T ratio was 10:1. Effector cells, activated PBLs; target cells, CNE2 cells; CNE2-CM, conditioned medium from CNE2 cells; Kyn, kynurenine.

Although TILs were often present in NPC specimens, they showed no evidence of anti-tumor activity at the tumor site, suggesting inhibition by the tumor tissue microenvironment. We used an *in vitro *assay to see if the cytolytic activity of lymphocytes was impaired by the tumor milieu created by expression of IDO. A standard LDH release assay was conducted using CNE2 cells as targets and PBLs stimulated by IL-2 for 96 h as effectors. PBLs lysed the target cells after IL-2 treatment, but the lysis rate was remarkably reduced by exposure to CNE2-CM from IFNγ-treated CNE2 cells (Fig. 5A). To further demonstrate the effect of IDO on the impaired cytotoxicity of PBLs, the specific IDO inhibitor, 1 MT, was used to block enzyme activity. Activated PBLs were cultured in CNE2-CM from CNE2 cells treated with or without 50 U/mL IFNγ and/or 100 μM 1 MT for 24 h, and the cytolytic activity was evaluated. The cytolytic activity of PBLs against CNE2 cells was functionally restored by the addition of 1 MT, compared with the groups cultured in the absence of 1 MT (Fig. 5B). HPLC analysis demonstrated that the addition of 100 μM 1 MT dramatically reduced Kyn production (Fig. 5C).

We also investigated if PBL cytotoxicity could be restored when IDO was removed. Activated PBLs were incubated with CNE2-CM derived from IFNγ-treated or untreated CNE2 cells containing 100 U/mL IL-2 for 96 h, and restored groups were then transferred to fresh medium containing 100 U/mL IL-2 and incubated for 24 h. The cytolytic activity of each group was analyzed by LDH release assay. PBLs cultured in CNE2-CM from IFNγ-treated CNE2 cells exhibited decreased cytolytic activity against target cells, compared with those in CNE2-CM from untreated CNE2 cells (Fig. 5D). However, the cytolytic activity of PBLs cultured in CNE2-CM derived from IFNγ-treated CNE2 cells was fully restored when the CNE2-CM was replaced by fresh medium for 24 h. In the presence of 100 μM 1 MT, both un-restored and restored groups maintained a similar level of cytolytic activity. These results suggest that the tumor milieu created by IDO-positive NPC cells could significantly impair the cytolytic function of activated lymphocytes, providing a potential mechanism for immune evasion in patients with NPC.

### Expression of IDO in tumor tissues from patients with NPC

**Figure 6:**
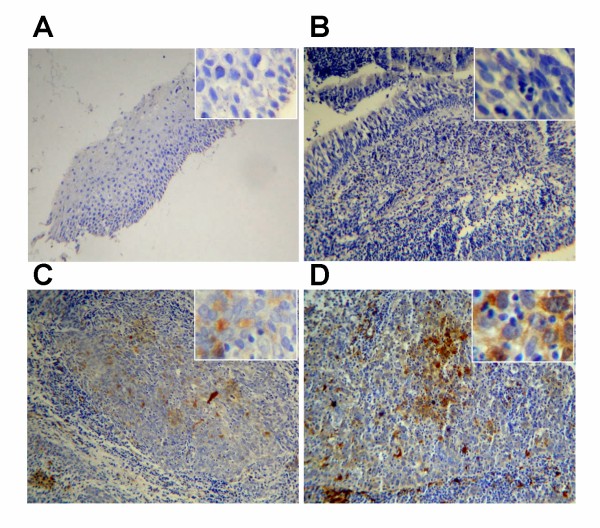
**Expression of indoleamine 2,3-dioxygenase (IDO) in nasopharyngeal carcinoma**. Normal nasopharyngeal tissue (A), tumor section from NPC type I (B), type II (C) and type III (D) were stained with antibodies to IDO, as described in Materials and Methods. Images were captured at ×100 magnification, and ×400 magnification for inserts.

In order to attain clinical evidence of IDO expression in NPC, the immunohistochemical assay was performed in NPC specimens and sections of normal nasopharyngeal tissues. The NPC specimens were stratified into three types according to WHO histological classification. No IDO-positive staining was observed among the epithelial cells or keratinizing cells in the sections of normal nasopharyngeal tissues (Fig. 6A) or NPC type I (Fig. 6B), whereas IDO-positive cells with typical nuclei were seen scattered in the sections of NPC type II (Fig. 6C) and III (Fig. 6D). In addition, infiltration of lymphocytes into the tumor tissues could be clearly identified in NPC type II and III, judged by morphology, consistent with the previously reported characteristics of NPC [25].

## Discussion

Unlike most carcinomas in which TILs are infrequently observed, NPC has intense lymphocyte infiltration in the tumor tissue [25,28], though these infiltrated lymphocytes apparently have limited ability to eliminate the tumor. The present study demonstrated, for the first time, that expression of IDO occurred in the NPC-derived cell line CNE2 and in tumor tissues from patients with NPC. Furthermore, we have shown that the expression of IDO in CNE2 cells was inducible by activated PBLs and that the cytolytic function of PBLs was impaired in the tumor milieu created by enzyme activity of IDO, providing a novel insight into the role of IDO in tumor immune evasion in NPC.

The infiltration of lymphocytes to tumor sites suggests that an immune response has been initiated, and Tang et al found that TILs in undifferentiated NPC are in an activated state [29]. However, NPC still progresses in most patients, and the prognosis of advanced stage NPC is frustratingly poor [30], indicating that tumor immune evasion occurs in these patients. Several mechanisms have been proposed to explain this e.g., the abnormal expression of IL-10 and Bcl-2 in NPC cells [31,32], as well as the impairment of IFNγ secretion and perforin expression in CD8^+ ^T cells [33]. In this study, we found that the immunosuppressive enzyme IDO was highly expressed in tumor tissues from NPC patients, suggesting that IDO-mediated immunosuppression may be involved in immune evasion, and so contribute to NPC progression.

Previous studies have demonstrated posttranslational control of IDO, implying that IDO expression and activity may not be correlated [34,35]. However, in the present study, analysis of Kyn production proved that the IDO expressed in CNE2 cells possessed functional enzymatic activity, and suggesting that active IDO was induced in NPC cells by IFNγ. We also showed that IFNγ induced IDO expression in CNE2 cells at concentrations as low as 5 U/ml, implying that IDO expression in NPC cells could be easily induced by low levels of IFNγ. The time course of IDO expression in CNE2 cells treated with IFNγ indicated that, once IDO protein was synthesized, it degraded slowly (>72 h), which could result in a long-lasting effect of IDO in NPC patients, resulting in severe tryptophan deprivation and Kyn accumulation at tumor sites. Hence, dense infiltration of lymphocytes or systemic administration of IFNγ could induce IDO expression and cause persistent, enhanced IDO activity in NPC patients.

Western blot analysis demonstrated that CM from activated PBLs was able to induce IDO expression in CNE2 cells. PBLs are known to functionally express multiple inflammatory cytokines, including IFNγ and TNFα, which are believed to be potent inducers of enzyme expression [36]. The ability of PBLs to induce IDO expression was also observed after culture in CNE2-CM derived from IDO-expressing CNE2 cells, suggesting that the IDO-generating condition did not directly impair cytokine release. This is in agreement with the results of previous studies, which found that the T cell activation markers, including IL-2, IFNγ, CD69, CD25, and CD71, were still expressed under conditions depleted of tryptophan and/or enriched in Kyn [13,37]. It has also been reported that merely activating the immune system, such as by adoptive T cell transfer using a conventional lymphoblastoid cell line-reactivated preparation [38,39], or by vaccination with dendritic cells [40,41], might be inadequate for immunotherapy, and may result in up-regulation of IFNγ and/or TNFα production, leading to IDO expression in tumor cells [41]. Further studies are needed to determine if IDO expression in CNE2 cells is directly induced by these cytokines, if these cytokines act synergistically to increase the expression of IDO, or if there is an independent pathway of IDO expression that exists in the CM from activated PBLs.

In the present study, we found that IFNγ-induced IDO expression in CNE2 cells did not promote apoptotic death of PBLs. IDO-expressing cells have been reported to promote apoptosis of blood T cells due to the cytotoxicity of tryptophan metabolites, such as Kyn, but not through tryptophan deficiency. Terness et al. and Frumento et al. showed that Kyn concentrations > 100 μM suppressed T cell proliferation by inducing cell death, whereas concentrations < 30 μM did not cause T cell apoptosis [26,42]. In the current study, IDO-expressing CNE2 cells generated Kyn in an IFNγ dose-dependent manner, and Kyn concentration in the cell culture system did not exceed 10 μM after treatment with 50 U/ml of IFNγ, which was too low to promote T cell death. Although it is difficult to extrapolate from *in vitro *to *in vivo *situations, the lack of a cytotoxic effect due to the presence of Kyn at a low concentration in the tumor microenvironment could explain previous observations of high densities of lymphocytes in NPC tumor tissue.

Once expressed in the tumor cells, IDO creates a local environment depleted of tryptophan and enriched in Kyn, thus rendering TILs susceptible to proliferation arrest and/or dysfunction. In our *in vitro *study, we found that exposure to CNE2-CM derived from IDO-expressing CNE2 cells dramatically weakened the cytolytic function of PBLs against target cells, but this attenuation could be reversed by addition of the IDO inhibitor, 1 MT. In this case, the down-regulation of the cytolytic function of PBLs did not appear to be due to the production of viral IL-10 (vIL-10), which was frequently observed in NPC tumor tissue [43], because vIL-10 was not detectable in CNE2 cells by reverse transcription polymerase chain reaction analysis (data not shown). These results suggest that PBLs are susceptible to the IDO-generating environment and that blocking IDO activity could restore their anti-tumor activity. We demonstrated that the cytolytic activity of PBLs could be fully restored by the replacement of CM from IDO-expressing CNE2 cells with fresh medium. Previous studies also found that IDO-mediated T cell proliferation arrest was overcome by the addition of tryptophan and removal of metabolites from the medium [9,26,42]. These observations suggest that the IDO-mediated suppression of PBL cytotoxicity is a reversible event, and that blocking IDO activity in tumor cells could help to re-establish the anti-tumor immune response, especially in tumors with high densities of infiltrating lymphocytes, such as NPC.

## Conclusion

In conclusion, we identified IDO expression in NPC and found that the enzyme could be efficiently induced by low dose IFNγ in the NPC cell line CNE2. PBLs exposed to the milieu generated by IDO-expressing CNE2 lost their cytolytic function, but did not undergo promoted cell death. We suggest that the negative effect of IDO expression in NPC cells on the cytolytic function of PBLs could contribute to the lack of efficacy of current immunotherapeutic strategies. Blocking both two isoforms of IDO, IDO1 and IDO2, by using their inhibitors L- and/or D-1-methyl-tryptophan [44] may provide a potential means of improving the outcomes of immunotherapy for the treatment of NPC.

## Abbreviations

NPC: nasopharyngeal carcinoma; IDO: indoleamine 2,3-dioxygenase; EBV: Epstein-Barr virus; Kyn: kynurenine; CNE2: human nasopharyngeal carcinoma cell line; PBMCs: Peripheral blood mononuclear cells; CM: conditioned medium; CNE2-CM: conditioned medium from CNE2 cells; HPLC: high-performance liquid chromatography; 1 MT: 1-methyl-D/L-tryptophan.

## Competing interests

The authors declare that they have no competing interests.

## Authors' contributions

PL and BX were responsible for most of the experimental work and drafted the manuscript. SC and JD participated in the design of this study. YH, GZ and YY assisted in the immunoassay detection. JD supervised this study, and involved in revising it critically for important intellectual content. All authors read and approved the final manuscript.

## Pre-publication history

The pre-publication history for this paper can be accessed here:

http://www.biomedcentral.com/1471-2407/9/416/prepub
